# Failure to detect Xenotropic murine leukaemia virus-related virus in Chinese patients with chronic fatigue syndrome

**DOI:** 10.1186/1743-422X-7-224

**Published:** 2010-09-13

**Authors:** Ping Hong, Jinming Li, Yongzhe Li

**Affiliations:** 1 Graduate School, Peking Union Medical College, Chinese Academy of Medical Sciences, Beijing, People's Republic of China; 2 National Center for Clinical Laboratories, Beijing Hospital, Beijing, People's Republic of China; 3 Peking Union Medical College Hospital, Beijing, People's Republic of China

## Abstract

**Background:**

Recent controversy has surrounded the question of whether xenotropic murine leukaemia virus-related virus (XMRV) contributes to the pathogenesis of chronic fatigue syndrome (CFS). To investigate the question in a Chinese population, 65 CFS patients and 85 blood donor controls were enrolled and multiplex real-time PCR or reverse transcriptase PCR (RT-PCR) was developed to analyze the XMRV infection status of the study participants. The assay was standardized by constructing plasmid DNAs and armored RNAs as XMRV standards and competitive internal controls (CICs), respectively.

**Results:**

The sensitivities of the multiplex real-time PCR and RT-PCR assays were 20 copies/reaction and 10 IU/ml, respectively, with 100% specificity. The within-run precision coefficient of variation (CV) ranged from 1.76% to 2.80% and 1.70% to 2.59%, while the between-run CV ranged from 1.07% to 2.56% and 1.06% to 2.74%. XMRV was not detected in the 65 CFS patients and 65 normal individuals out of 85 controls.

**Conclusions:**

This study failed to show XMRV in peripheral blood mononuclear cells (PBMCs) and plasma of Chinese patients with CFS. The absence of XMRV nucleic acids does not support an association between XMRV infection and the development of CFS in Chinese.

## Background

Chronic fatigue syndrome (CFS) is a multisystem disease which is characterized by severe and debilitating fatigue, abnormal sleep behaviour, impaired memory and concentration, and musculoskeletal pain [1]. The constellation of symptoms is non-specific and can be caused by a variety of diseases. Studies have identified various features relevant to the pathogenesis of CFS, such as viral infection, abnormal immune function, exposure to toxins, chemicals and pesticides, stress, hypotension, abnormal lymphocyte levels, and neuroendocrine dysfunction. However, the precise underlying mechanisms of the disease and the means by which they interact in patients with CFS remain to be clarified [2].

Recent works have emphasized the frequent association of CFS with gammaretrovirus (XMRV) infection, although the role of XMRV as a causative agent of CFS remains controversial. In a recent US study, Lombardi et al [3] found that about 67% (68/101) of patients with CFS carried detectable levels of XMRV DNA in their peripheral blood mononuclear cells (PBMCs). Moreover, replicating virus was isolated from stimulated PBMCs in vitro. If confirmed, these findings would have a serious impact on understanding the pathogenesis of this complex and debilitating disease. However, another 3 recent reports showed that XMRV was absent in PBMCs from European CFS patients [4-6]. The results were similar in a recent report from the US [7], leading to an intense scientific debate over the relationship between the virus and CFS [8]. It is not yet clear whether the distribution of this virus is primarily dependent on geographic restrictions or, more likely, the diagnostic techniques used, such as PCR and real-time PCR. In terms of methodology, one of the risks associated with testing samples by PCR is the frequent occurrence of false negatives as a result of PCR inhibition [9]. The inclusion of an internal control (IC) serves as a monitor for false negatives caused by DNA degradation or inhibitory factors that may be present in clinical samples [10]. In previous studies of XMRV detection [4-7], non-competitive ICs, such as GAPDH, were used to monitor for false-negative reactions. In the non-competitive IC strategy, separate primer pairs are used to detect the IC and the target nucleic acid. Nevertheless, the non-competitive ICs may introduce different amplification efficiencies due to their natural inter-individual variation, and may produce false-negative results. This matter is of considerable importance in the extensive controversy surrounding XMRV detection in CFS patients.

A review of the diverse results from previous studies reveals several questions about worldwide distribution and whether the retrovirus is linked to CFS, at all. Until now, no information has been published regarding XMRV infection in Chinese CFS sufferers. We developed sensitive multiplex real-time PCR and reverse transcriptase PCR (RT-PCR) assays, using a competitive internal control (CIC) strategy to ensure PCR integrity and eliminate false-negative results, to detect XMRV proviral DNA and viral RNA, respectively, in the PBMCs and plasma of Chinese CFS patients. The assays were standardized using constructed XMRV DNA or armored RNA standards, and their performances were evaluated.

## Materials and methods

### Study subjects and samples

Sixty-five CFS patients and 85 blood donors, including 65 healthy controls and 20 controls with hepatitis B, hepatitis C, human immunodeficiency virus type 1 infection, or human T-cell leukaemia virus infection (confirmed at the blood bank) were enrolled. Patients and controls were closely matched for age, sex, and place of residence. Both groups were aged between 20 and 55 years, and the ratios of women to men were 35:30 (CFS) and 44:41 (blood donors). Samples were collected from 2007 to 2009. CFS patients were recruited from clinics in Peking Union Medical College Hospital. All patients underwent full medical and neurological evaluations and were tested to exclude alternative explanations for their symptoms. Additionally, they fulfilled the 1994 international research criteria for diagnosis of CFS [1], which requires the presence of fatigue with 4 or more additional symptoms and was established to help distinguish CFS from other illnesses that cause fatigue. Blood donors were enrolled from the Beijing blood centre. All subjects provided informed consent prior to their participation in the study.

Whole blood was obtained by venipuncture from 85 blood donors and 65 CFS patients. PBMCs and plasma were isolated immediately by Ficoll-Hypaque-1077 (Sigma) from whole blood and stored at -80°C within 2 hours of sampling.

### DNA and RNA preparation

DNA from 100 μl PBMCs (about 5.0 × 10^2 ^to 2 × 10^3 ^cells) or RNA from 140 μl plasma was isolated according to the manufacturer's instructions (QIAamp DNA Blood Mini Kit, QIAamp Viral RNA Mini kit, QIAGEN GmbH, Germany); extracted DNA was eluted in 100 μl DNAse-free water, while RNA was eluted in 60 μl RNase-free water. Both were immediately stored at -80°C.

### Primers and probes

Primers and probes for the XMRV real-time detection assay were designed to amplify regions of the XMRV *gag *gene (nt 462-523). Primer and probe sequences were optimized using Primer Express (Applied Biosystems) and were synthesized as previously described [11], to detect both XMRV proviral DNA and XMRV viral RNA. In order to calibrate the constructed armored RNAs to an international (IU) value, primers were designed to amplify regions of the HCV 5' UTR. Probes for the detection of XMRV and CIC were 5'-labelled with 6-carboxyfluorescein (FAM) or 6-carboxyhexachlorofluorescein (HEX), and all were 3'-labelled with Black Hole Quencher Dye (BHQ). The sequences and characteristics of the primers and probes are listed in Table 1.

**Table 1:** Primer and probe sequences

Primer or probe	Sequence (5'-3')
Gag-1S	5'-TTGGCCGGCCACATGAGGATCACCCATGTCGTGTTCCCAATAAAGCCTTTTGCTGTTTG-3'
Gag-1A	5'-ATTCAGACGGGGGCGGGAATGTCGGCTTTGAGGGGGCCTGAGTGTCTCTGTCTCTCGTC-3'
Gag-2S	5'-GACGAGAGACAGAGACACTCAGGCCCCCTCAAAGCCGACATTCCCGCCCCCGTCTGAAT-3'
Gag-2A	5'-GAGTGATCTATGGTGGAGACATGGGTGATCCTCATGTGCCGCCTCTTCTTCATTG-3'
HCV- S	5'-CAATGAAGAAGAGGCGGCACATGAGGATCACCCATGTCTCCACCATAGATCACTC-3'
HCV-A	5'-CCTTAATTAAACATGGGTGATCCTCATGTGGTTGGTGTTACGTTTGGTT-3'
Gag-3S	5'-GGACTTTTTGGAGTGGCTTTGTT-3'
Gag-3A	5'-GCGTAAAACCGAAAGCAAAAAT-3'
Gag p	FAM5'-ACAGAGACACTTCCCGCCCCCG-3'BHQ
IC p	HEX5'-CAGGCCCCCTCAAAGCCGACAT-3'BHQ
β-actin A	5'-CCTGGCACCCAGCACAAT-3'
β-actin S	5'-GCTGATCCACATCTGCTGGAA-3'
β-actin p	FAM5'-ATCAAGATCATTGCTCCTCCTGAGCGC-3'TAKARA

*Fse*I and *Pac*I restriction enzyme sites are underscored; a C-variant *pac *site is indicated by boldface type. The broken line indicates the internal control sequences inserted in the CIC. FAM: 6-carboxyfluorescein; HEX: 6-carboxyhexachlorofluorescein; BHQ: Black hole quencher dye.

### Preparation of the XMRV DNA standard and the CIC: recombinant plasmids pACYC-MS2-2V and pACYC-MS2-IC-2V

An exogenous chimeric sequence 1584 bp in length was inserted into pACYC-MS2 [12] (previously constructed by our laboratory) with three C-variant *pac *sites inserted at the beginning, middle, and end. This sequence was obtained by overlapping extension PCR [11,12] amplification of XMRV (nt 33 to 1149, 1117 bp amplified from plasmid VP62 [3], kindly provided by Lombardi; [Genbank: EF185282]) and HCV (nt 25 to 445, 420 bp amplified from pNCCL-HCV [13], constructed by our laboratory; [Genbank: AF139594]). The primers used in this method are shown in Table 1.

**Figure 1:**
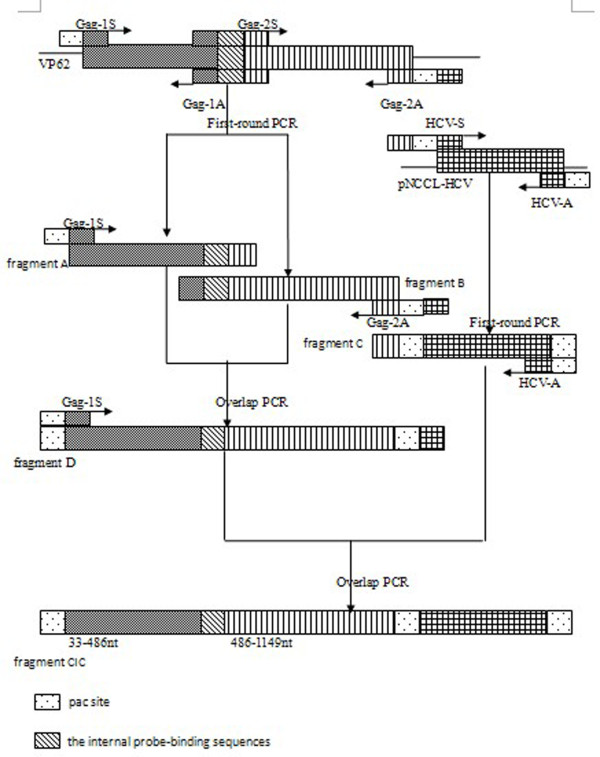
**Construction of CIC by overlapping PCR**. During the first-round of PCR, 3 fragments (A, B and C: VP62 33-486 nt, VP62 486-1149, and the HCV 5'UTR) were amplified from plasmid VP62 (kindly provided by Lombardi) and pNCCL-HCV (constructed by our laboratory) using primers Gag-1S and Gag-1A, Gag-2S and Gag-2A, and HCV-S and HCV-A. In the first overlapping PCR, fragment D was amplified from fragments A and B using primers Gag-1S and Gag-2A; the internal probe-binding sequences were introduced into fragment D. Fragment CIC was obtained by a second overlapping PCR using primers Gag-1S and HCV-A to amplify from fragments D and C.

CIC sequences were identical to the 1584-bp exogenous chimeric sequence, except for the probe-binding sites which were replaced by internal probe sequences using overlapping extension PCR [14]. The sequence of these 22 artificial random nucleotides shared a similar nucleotide composition as the wild type XMRV probe (Fig. 1).

The resulting recombinant plasmids pACYC-MS2-2V and pACYC-MS2-IC-2V were validated by sequencing. The concentrations of DNA standard and CIC were assessed by UV-spectrophotometry and DNA copy numbers were calculated.

### Preparation of the viral RNA standard and its CIC (armored RNAs)

Both pACYC-MS2-2V and pACYC-MS2-IC-2V were transformed into *E. coli *strain BL21 (DE3). The armored RNAs were expressed, purified, and verified [12,13], and their stabilities were also verified [12,13].

The purified armored RNAs were calibrated against the World Health Organization (WHO) Second International Standard for HCV RNA (National Institute for Biological Standards and Controls [NIBSC], code 96/798, UK), using an HCV RNA PCR fluorescence quantitative diagnostic kit (Shanghai, Kehua) [13]. The samples were tested in triplicate and the quantification values were averaged. The concentration of the CIC was evaluated by the same method.

### Multiplex real-time PCR and RT-PCR for XMRV detection

Standard DNA and armored RNA were quantified, diluted to obtain 10-10^6 ^copies/μl and IU/ml, respectively, and used to determine the linearity, sensitivity, specificity, and reproducibility of the multiplex real-time assays. They also served as external positive controls (EPCs) in the multiplex real-time PCR and RT-PCR assays.

To determine the optimal CIC concentration for the real-time assay, a chequer-board assay was performed in which XMRV standards (10^5 ^to 10^2 ^DNA copies/μl or 10^5 ^to 10^2 ^RNA IU/ml) were spiked with 3 different concentrations (10^5 ^to 10^3 ^copies/μl or IU/ml, respectively) of the CIC, and the template mixture was assayed. Thereafter, it was coamplified or coextracted and coamplified with the samples in the same reaction tube. The final optimized PCR mixture (25 μl) contained 12.5 μl QuantiTect Probe PCR or RT-PCR Master Mix (QIAGEN, QuantiTect Multiplex PCR or RT-PCR kit), 0.4 μM XMRV-specific primers, 0.4 μM XMRV-specific probes, and 0.2 μM IC-specific probe, 8.3 μl sample (2.0 μl of XMRV DNA, 1.0 μl CIC DNA(1000 copies/μl added during the amplification step), and 5.3 μl DEPC-treated water ) or 0.25 μl QuantiTect RT Mix, 10 μl RNA (1000 IU/ml armored RNA CIC added to each sample prior to extraction). PCR was performed with an ABI 7500 sequence detection system as follows: an initial denaturation step at 95°C for 15 min, 45 cycles at 94°C for 15 s and 60°C for 1 min. In addition, the RT-PCR included an initial reverse transcription step of 50°C for 30 min.

The linearity and sensitivity of the XMRV assay were determined by using a dilution series of the DNA or armored RNA standard (10 copies to 10^6 ^copies/μl or IU/ml, respectively) in PBMCs DNA or plasma from a healthy donor. To mimic the conditions of the multiplex real-time PCR or RT-PCR procedures, we also included a steady concentration of CIC (1000 copies/μl or IU/ml, respectively). Experiments were performed in triplicate at each concentration.

Forty-five controls, which included 20 subjects with hepatitis B, hepatitis C, human immunodeficiency virus type 1 infection, or human T-cell leukaemia virus infection and 25 out of 65 healthy controls, were used to determine the specificity of the real-time assay.

The within-run precision of the quantitative real-time assay was assessed by evaluating 10 replicates of 3 dilutions of the DNA plasmid or armored RNA standard (10^5^, 10^4^, and 10^2 ^copies/μl or IU/ml, respectively), while the between-run precision was assessed by testing the same samples 10 times in 10 separate experiments. The coefficients of variance (CV) of the threshold cycles (Ct) were calculated.

Samples from 65 CFS cases and 65 healthy controls were tested using the standard curve method by multiplex real-time PCR or RT-PCR with CICs (10^3 ^copies/μl or IU/ml, respectively, present in each sample). The standard curves were generated from serial dilutions of the standards (10^6 ^to 10^2 ^copies/μl or IU/ml, respectively). In addition, pACYC-MS2-2V or armored RNA standard was used as an EPC. To control for the integrity of the DNA or RNA, the cellular β-actin gene was amplified in all clinical specimens and was tested under the same conditions, but with 0.4 μM β-actin specific primers and 0.4 μM β-actin specific probe (5' FAM, 3' TAKARA-labelled) (see Table 1).

## Results

### Construction of XMRV DNA standard and CIC plasmid

**Figure 2:**
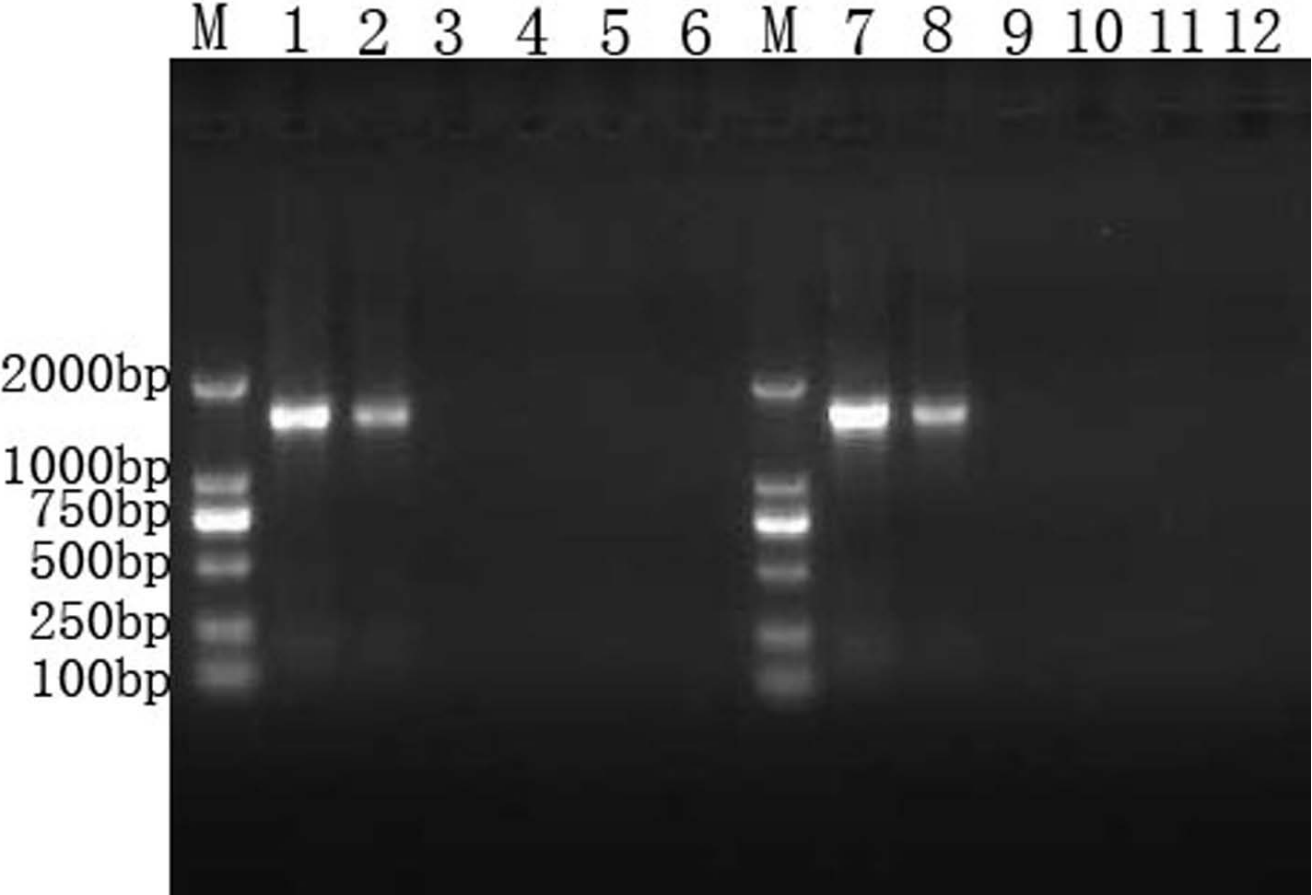
**Gel electrophoresis of PCR and RT-PCR products**. Lane M, DNA marker; Products amplified from pACYC-MS2-2V are represented in lanes 1-6: lane1, positive control; lane 2, RT-PCR product of RNA extracted from armored RNA; lanes 3-6, the 4 negative controls (H_2_O, H_2_O after extraction and RT, RNA extracted from armored RNAs without RT, and armored RNAs without extraction and RT); lanes 7-12 represent the same treatments, but amplified from pACYC-MS2-IC-2V.

The PCR amplification products from the DNA standard or CIC plasmid (using primer Gag-1S and HCV-A) were full length (1584 bp and 1606 bp, respectively). Sequencing demonstrated that the exogenous chimeric sequences were successfully inserted into the pACYC-MS2 vector. The PCR products were analyzed by electrophoresis on an agarose gel (1%) (Fig. 2).

### Preparation of the viral RNA standard and its internal control (armored RNAs)

**Figure 3:**
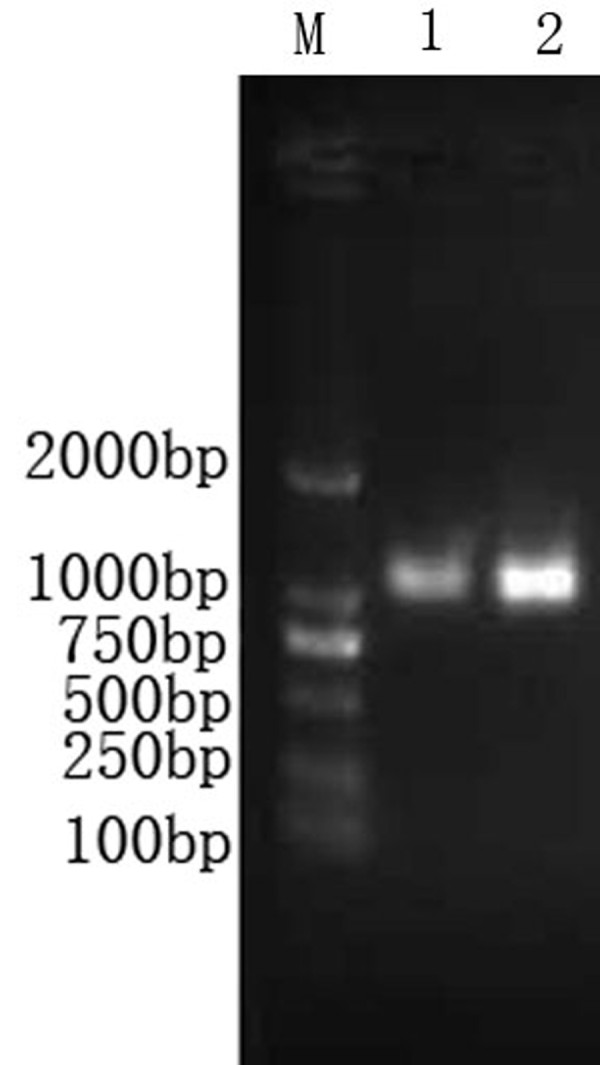
**Electrophoresis of armored RNAs after purification by gel exclusion chromatography**. Lane M, DNA marker; lane 1, armored RNA standard; lane 2 armored RNA CIC **(1% agarose gel )**.

The purified armored RNAs were electrophoresed and a single band of approximately 1.0 kb could be seen by agarose gel analysis (Fig. 3). The RT-PCR amplification products of the RNA extracted from armored RNAs were analyzed by agarose gel electrophoresis (Fig. 2). PCR products were then verified by sequencing.

Armored RNAs in EDTA-preserved human plasma incubated at 4°C, 37°C, and room temperature were stable for over 3 months (data not shown).

To evaluate the performance characteristics of armored RNA as a calibrator for XMRV RNA assays, we used the HCV international standard to calibrate serially diluted armored RNA. The concentrations of the chimeric armored RNA for the 5 dilutions (10^6^, 10^5^, 10^4^, 10^3^, and 10^2^) were 5.63 × 10^6 ^IU/ml, 6.01 × 10^5 ^IU/ml, 5.47 × 10^4 ^IU/ml, 5.36 × 10^3 ^IU/ml, and 5.75 × 10^2 ^IU/ml. The concentrations of the CIC were evaluated in the same way, at 1.12 × 10^6 ^IU/ml, 9.78 × 10^4 ^IU/ml, 1.03 × 10^4 ^IU/ml, 1.15 × 10^3 ^IU/ml, and 1.07 × 10^2 ^IU/ml.

### Multiplex real-time PCR and RT-PCR for XMRV detection

A dilution series of the DNA or RNA XMRV standard was spiked with 3 different concentrations (10^5 ^to 10^3 ^copies/μl or IU/ml) of the CIC and used as a mixed template. We determined that 1000 copies/μl of DNA plasmid or 1000 IU/ml armored RNA was the optimal CIC concentration for the multiplex real-time assay (Table 2).

**Table 2:** Optimization of CIC concentration

(1) Multiplex real-time PCR									
**pACYC-MS2- IC-2V plasmid CIC concentration (copies/μl)**	**pACYC-MS2-2V plasmid standard 1 × 10**^**5 **^**copies/μl**		**pACYC-MS2-2V plasmid standard 1 × 10**^**4 **^**copies/μl**		**pACYC-MS2-2V plasmid standard 1 × 10**^**3 **^**copies/μl**		**pACYC-MS2-2V plasmid standard 1 × 10**^**2 **^**copies/μl**		**XMRV 0 copies/μl**
	
	**CIC (HEX)Ct**	**XMRV (FAM)Ct**	**CIC (HEX)Ct**	**XMRV (FAM)Ct**	**CIC (HEX)Ct**	**XMRV (FAM)Ct**	**CIC (HEX)Ct**	**XMRV ((FAM)Ct**	**CIC (HEX)Ct**

100000	39.22	28.79	32.18	31.58	28.92	34.81	28.50	No Ct	28.34
10000	37.85	28.12	31.26	31.06	32.36	34.56	31.45	44.26	31.55
1000	39.52	24.73	37.20	27.76	31.50	31.18	35.79	34.28	34.11
0	No Ct	24.69	No Ct	28.47	No Ct	30.73	No Ct	33.81	No Ct

**(2) Multiplex real-time RT-PCR**									

**Armored RNA concentration (IU/ml)**	**Armored RNA standard 1 × 10**^**5 **^**IU/ml**		**Armored RNA standard 1 × 10**^**4 **^**IU/ml**		**Armored RNA standard 1 × 10**^**3 **^**IU/ml**		**Armored RNA standard 1 × 10**^**2 **^**IU/ml**		**XMRV 0 IU/ml**
	
	**CIC (HEX)Ct**	**XMRV (FAM)Ct**	**CIC (HEX)Ct**	**XMRV (FAM)Ct**	**CIC (HEX)Ct**	**XMRV (FAM)Ct**	**CIC (HEX)Ct**	**XMRV (FAM)Ct**	**CIC (HEX)Ct**

100000	39.50	25.24	30.89	28.28	27.12	No Ct	26.35	No Ct	26.07
10000	37.96	25.00	30.70	28.22	30.37	31.17	27.79	37.06	29.49
1000	No Ct	25.25	30. 65	28.03	33.18	31.14	33.87	34.40	33.27
0	No Ct	25.18	No Ct	27. 82	No Ct	30.78	No Ct	37.11	No Ct

Ct values indicate the standard concentrations. A sample with Ct > 45 cycles was considered to be negative. Concentrations of XMRV DNA/RNA and pACYC-MS2-2V plasmid/armored RNA standard were indicated by FAM and HEX signals, respectively.

**Figure 4:**
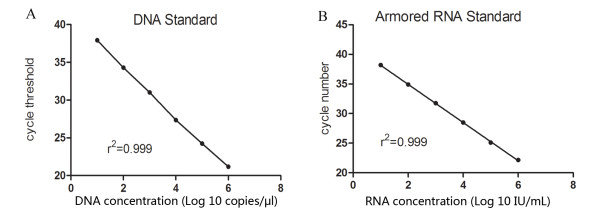
**Linearity and sensitivity of the DNA or armored RNA standards in the multiplex real-time assay**. Standard curves of the DNA standard **(A) **and RNA standard **(B) **were linear. Amplification of ten-fold serial dilutions from 10^6 ^copies/μl to 10 copies/μl or 10^6 ^IU/ml to 10 IU/ml of the standard demonstrated a standard curve R^2 ^of 0.999, which was yielded by plotting the Ct values against the log_10 _XMRV DNA or RNA standard concentration.

Linear regression analysis of the Ct values against the log_10 _XMRV DNA or armored RNA standard concentration yielded r^2 ^= 0.999. The lowest detectable concentration of XMRV DNA or armored RNA standard was 20 copies/reaction, calculated as 10 copies/μl × 2.0 μl XMRV DNA standard per reaction, or 10 IU/mL, respectively (Fig 4).

The specificity of the multiplex real-time assay was 100% in testing of the 45 controls.

Reproducibility was established based on the Ct values obtained within each run (within-run) and between runs. The within-run precision CV ranged from 1.76% to 2.80% or 1.70% to 2.59%, while the between-run CV ranged from 1.07% to 2.56% or 1.06% to 2.74% (Table 3).

**Table 3:** Reproducibility of the real-time PCR/RT-PCR assay

Reproducibility	XMRV DNA/RNA (copies/μl/IU/ml)	Number of determinations	Mean Ct (DNA/RNA)	SD (DNA/RNA)	CV(%) (DNA/RNA)
within-run	10^5^	10	24.47/25.13	0.50/0.52	2.03/2.06
	10^4^	10	27.25/27.99	0.48/0.48	1.76/1.70
	10^2^	10	33.81/34.62	0.94/0.90	2.80/2.59
between-run	10^5^	10	24.97/26.17	0.28/0.32	1.14/1.23
	10^4^	10	27.98/29.14	0.30/0.31	1.07/1.06
	10^2^	10	34.43/35.94	0.88/0.99	2.56/2.74

**Figure 5:**
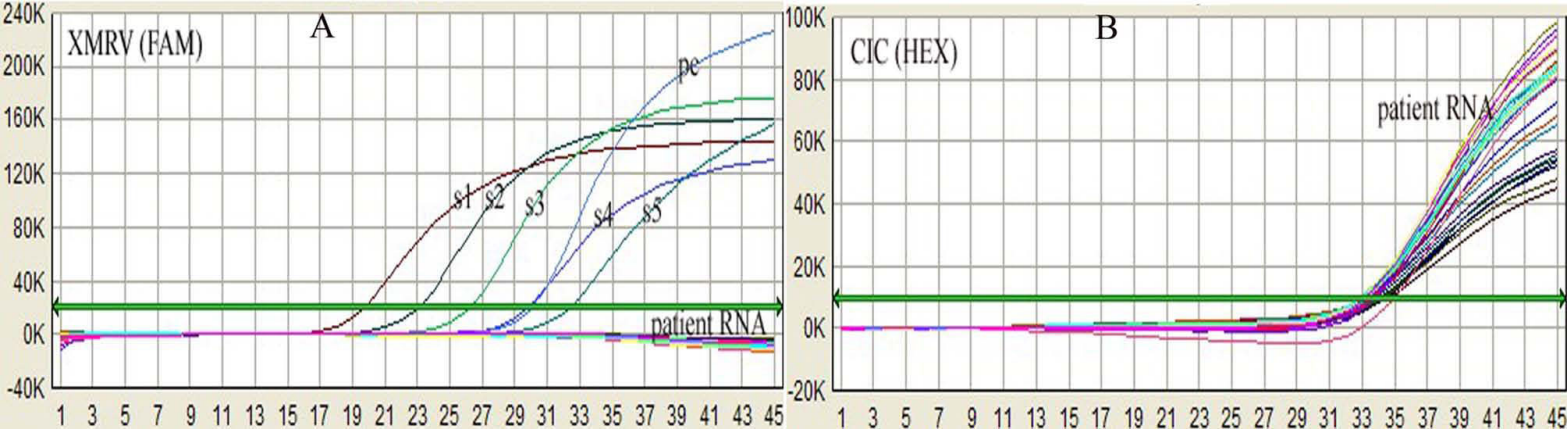
**Multiplex real-time assay for detection of patient XMRV proviral DNA or viral RNA **Examples of viral RNA screen of 25 CFS patient plasma samples including CICs in each reaction. (A) Amplification plot for a plasma sample obtained from a CFS patient: No XMRV-specific signals were detected. S1-S5 were armored RNA standards (from 10^7 ^to 10^3 ^IU/ml), pc was an armored RNA EPC. (B) Amplification plot of the CICs (1000 IU/ml), which were added to patient samples.

The amounts of XMRV DNA derived from PBMC and RNA derived from plasma were determined by using the standard curve method. No signals for the XMRV-specific probe were detected, while all 65 CFS cases and 65 healthy controls generated positive CIC (1000 copies/μl or IU/ml, respectively) signals with Ct values between 32 and 36 (Figure 5). The β-actin gene was detected in all clinical specimens.

## Discussion

Sensitive multiplex real-time PCR and RT-PCR assays with CICs were established to detect XMRV proviral DNA in PBMCs or viral RNA in plasma, respectively, from Chinese patients with CFS. The virus was not detected in any of our study subjects; these results do not support an association between XMRV and CFS in Chinese.

Our findings appeared to be inconsistent with a previous report of XMRV DNA isolation from PBMCs of CFS patients in the US [3]. Technical differences can be ruled out as a reason for the failure to detect XMRV. The sensitivity of the multiplex real-time PCR (20 copies/reaction) was likely to be as sensitive, if not more so, as the end point PCR method previously used [3], thus suggesting that multiplex real-time PCR can be used for the detection of XMRV proviral DNA. The end-point PCR method used in the previous study requires multiple manipulations of the sample after the amplification step, thus increasing the risk of carryover contamination. The possibility that proviral DNA degradation during the extraction process may have led to our negative results seems unlikely. The β-actin gene was positive for all clinical specimens, confirming the integrity of the DNA. In addition, samples used in this research were representative of typical patients with CFS, which met the 1994 Centers for Disease Control and Prevention case definition of CFS (called the Fukuda criteria). Although the patients studied by Lombardi et al [3] were reported to fulfil the same criteria, a clear description of their patient and control cohorts was lacking.

Several PCR-based methodologies have been developed for the detection of XMRV DNA, including end point and real-time PCR methodologies [4-7]. Beta-globin gene, GAPDH, and β-actin were used as non-competitive ICs to validate DNA integrity in 4 recent studies of XMRV [4-7]. However, DNA concentrations may vary widely between clinical samples. Van Kuppeveld et al [6] amplified a known amount of phocine distemper virus (PDV) that had been added to clinical samples to monitor RNA quality and to detect amplification inhibition. Although attractive, the use of live viruses as internal controls may raise concerns regarding safety and consistency between preparations. Additionally, the performance of non-competitive ICs is imperfect due to differences in the amplification efficiencies of different target nucleic acids [15]. Here, we used CIC as a substitute. The CIC was a constructed plasmid which mimicked the template with the same length and primer binding sites, and similar GC content. In order to avoid suppression of target amplification and possible competition between the target and CIC, we optimized the concentration of CIC added to the samples. IC concentrations of more than 1000 copies/μl altered the Ct of almost all standards which yielded an underestimation of the concentration. CIC at 1000 copies/μl was determined to be optimal for our real-time assay. Our results indicated that no inhibitory effects were at play in the multiplex real-time assay we used to screen our study population.

The pathogenesis and outcome of XMRV infection may be associated or even causally linked with plasma viral RNA loads, as well as proviral loads. In addition to XMRV proviral DNA detection, we developed a sensitive multiplex real-time RT-PCR assay to detect XMRV viral RNA in plasma. We constructed armored RNAs to serve as the XMRV viral RNA standard and CIC to evaluate the analytical linearity, sensitivity, specificity, and reproducibility of the detection assay. Both were stable in normal human EDTA-preserved plasma at 4°C, 37°C, and room temperature for over 3 months. Armored RNA is a kind of non-infectious recombinant virus-like particle (VLP) containing target exogenous RNA. In comparison to naked RNA, as armored RNA is a more suitable candidate for a positive control or standard in the quantification of RNA viruses, because it is RNase-resistant, stable, non-infectious, and easily extracted by conventional methods [16-19]. Moreover, armored RNA can serve as a control for all stages of the RT-PCR assay, from extraction through amplification. The inclusion of the HCV 5'UTR made it easy to assign an IU value to the XMRV RNA and the CIC within the armored RNA, avoiding the necessity for the complex procedures involved in value assignment of calibrators or standards when international standards (IS) are not available [12]. These characteristics of armored RNA ensure the validity of our data. Conflicting results have made the associations between XMRV and CFS unclear; it is therefore important to produce a 'universal' XMRV standard so that the results of different assays may be compared. By using an armored RNA standard, different research groups and clinical laboratories may directly compare their quantitative data. Nevertheless, we did not detect XMRV viral RNA with our armored RNA-standardised method in plasma samples from a Chinese study population.

These findings may not be generalisable beyond the study population because XMRV infection rates may vary geographically. Similarly, although XMRV was initially discovered in tumour tissues of a subset of patients with prostate cancer [20], other studies have shown a variable incidence of the virus in prostate tumours. One US study found XMRV in up to 23% of prostate cancer tumours [21]; however, a recent German study found a 0% incidence of XMRV [22]. Additional research is needed to determine what, if any, role XMRV plays in CFS in Chinese patients.

## Conclusions

This study failed to show the presence of XMRV in PBMCs and plasma of Chinese patients with CFS using the multiplex real-time PCR assay; these results do not support an association between XMRV and CFS in people of Chinese ancestry.

## Competing interests

The authors declare that they have no competing interests.

## Authors' contributions

PH planned the experimental design and drafted the manuscript. JL generated the concept for the study, participated in its design and coordination, and helped to revise the manuscript. YZL collected specimens and data from the study population. All authors read and approved the final manuscript.
